# Comparison of reconstruction and acquisition choices for quantitative T2* maps and synthetic contrasts

**DOI:** 10.1016/j.ejro.2018.12.006

**Published:** 2018-12-31

**Authors:** Riikka Ruuth, Linda Kuusela, Teemu Mäkelä, Susanna Melkas, Antti Korvenoja

**Affiliations:** a HUS Medical Imaging Center, Radiology, University of Helsinki and Helsinki University Hospital, P.O. Box 340, FI-00029, HUS, Finland; b Department of Physics, Faculty of Science, University of Helsinki, P.O. Box 64, FI-00014, Helsinki, Finland; c Clinical Neurosciences, Neurology, University of Helsinki and Helsinki University Hospital, P.O. Box 302, FI-00029, HUS, Finland

**Keywords:** Magnetic resonance imaging, Image quality, Quantitative MRI, MRI reconstruction, T2* mapping, Synthetic contrasts

## Abstract

**Aim and scope:**

A Gradient Echo Plural Contrast Imaging technique (GEPCI) is a post-processing method, which can be used to obtain quantitative T2* values and generate multiple synthetic contrasts from a single acquisition. However, scan duration and image reconstruction from k-space data present challenges in a clinical workflow. This study aimed at optimizing image reconstruction and acquisition duration to facilitate a post-processing method for synthetic image contrast creation in clinical settings.

**Materials and methods:**

This study consists of tests using the American College of Radiology (ACR) image quality phantom, two healthy volunteers, four mild traumatic brain injury patients and four small vessel disease patients. The measurements were carried out on a 3.0 T scanner with multiple echo times. Reconstruction from k-space data and DICOM data with two different coil-channel combination modes were investigated. Partial Fourier techniques were tested to optimize the scanning time.

**Conclusions:**

Sum of squares coil-channel combination produced artifacts in phase images, but images created with adaptive combination were artifact-free. The voxel-wise median signed difference of T2* between the vendor’s adaptive channel combination and k-space reconstruction modes was 2.9 ± 0.7 ms for white matter and 4.5 ± 0.6 ms for gray matter. Relative white matter/gray matter contrast of all synthetic images and contrast-to-noise ratio of synthetic T1-weighted images were almost equal between reconstruction modes. Our results indicate that synthetic contrasts can be generated from the vendor’s DICOM data with the adaptive combination mode without affecting the quantitative T2* values or white matter/gray matter contrast.

## Highlights

• Phase images have artifacts if reconstructed with a vendor’s sum of squares mode.
• Quantitative T2* values can be obtained from DICOM data instead of k-space data.
• Reconstruction from DICOM data does not reduce white matter/gray matter contrast.

## 1 Introduction

The Gradient Echo Plural Contrast Imaging technique (GEPCI) can be used to obtain quantitative T2* values and multiple synthetic contrasts from a single multi-echo gradient echo acquisition [1]. The clinical potential of this imaging method has been explored in multiple sclerosis [[2], [3], [4]], Alzheimer’s disease [5] and psychiatric diseases [6]. Potentially these synthetic contrasts could find applications in imaging of brain trauma or small vessel disease as susceptibility weighted images (SWI), T1-weighted images, and T2* contrasts obtainable with GEPCI post-processing are commonly used in these clinical settings [7].

The GEPCI technique requires images to be reconstructed for ten echo times (TE) and each coil-channel separately, while the acquisition must be performed at a clinically desirable resolution and coverage. When setting up the imaging protocol for the 32-channel head coil, we noticed that reconstructing DICOM images for each channel separately was not possible with our scanner, due to insufficient computing power and storage space. Thus, the data required for GEPCI post-processing had to be saved as k-space in vendor specific data format. An acquisition with a 32-channel head coil, resolution of 1.0 × 1.0 × 2.0 mm³, field of view (FOV) 256 mm × 192 mm, and 64 slices results in 21 GB of raw data. Such a large amount of data complicates the offline reconstruction by requiring considerable network and storage capacity. Similar issues can be encountered also on scanners from other vendors. Starting the post-processing by using coil-combined DICOM images alleviates these practical issues, as the generated data are less than 2% of the original k-space data size. The use of DICOM images would also facilitate management and transfer of the data in a hospital environment.

The relatively long 13-minute whole head acquisition time is also demanding for the patients. Shorter acquisition times are always sought after, and the partial Fourier [8] is a way to reduce the acquisition time. The use of k-space data can complicate the utilization of typical sequence speed-up techniques, when the reconstruction algorithms must take into account the undersampling of k-space [8] and coil sensitivity profiles [9].

These obstacles to the use of multi-contrast imaging must be overcome if this modality is to be adopted in clinical settings. We therefore seek a reasonable balance between technical image quality, acceptable imaging time, and moderate data storage demands. The purpose of this study was to assess the impact of channel combination method and partial Fourier technique on the image quality, relaxation times, and the contrast in synthetic images.

## 2 Material and methods

The studies were approved by the ethics committee of the Hospital District of Helsinki and Uusimaa and consisted of phantom, volunteer, and patient acquisitions. The phantom study was performed to assess the impact of combination modes and acquisition parameters on the image quality. Volunteer studies were performed to investigate different acquisition settings. Patients were imaged as parts of larger research projects, and they were included to gain insight into the impact of different approaches in a clinical setting.

### 2.1 Acquisition

The American College of Radiology (ACR) image quality phantom [10], two healthy volunteers, four mild traumatic brain injury patients, and four small vessel disease patients were scanned using a Siemens Verio 3.0 T (Erlangen, Germany) MRI system. In all measurements, a 3D multi-echo gradient-recalled echo sequence with a flip angle 30°, TR 49 ms and 10 TE 4–40 ms with ΔTE 4 ms was used.

The ACR phantom measurements were performed with a 12-channel head coil, because the phantom did not fit into the 32-channel head coil. The images were acquired with a 1 ͯ 1 mm² in-plane resolution and slice thicknesses of 2 mm and 5 mm, where the FOV was 256 mm × 216 mm and 256 mm × 200 mm, respectively. The data with 5 mm slice thickness was acquired, because the ACR phantom and measurements are designed particularly for this thickness. One additional acquisition was performed with FOV 256 mm × 216 mm and slice thickness of 1 mm to get more precise estimates of T2* values and best possible image quality. To assess the impact of partial Fourier imaging speedup techniques, acquisitions with partial Fourier in phase encoding direction (PFP) and/or slice encoding direction (PFS) were carried out with k-space coverage factors 6/8, 7/8, and full coverage.

To further assess the usability of the partial Fourier technique, one volunteer was imaged with the 12-channel head coil and the other with the 32-channel head coil. The GEPCI sequence in all brain imaging was acquired with a resolution of 1 × 1×2 mm³, FOV 256 mm × 192 mm and 64 slices. For the volunteer measurements, the PFP and PFS combinations were excluded to reduce the total scanning time.

All patient data were acquired with a 32-channel head coil, and only one sequence without partial Fourier technique was obtained for each patient. A sagittal T1-weighted 3D-MPRAGE image with isotropic 1 mm³ resolution, TR 1900 ms, TE 2.5 ms, TI 900 ms and FOV 250 mm ͯ 250 mm was acquired from the patients for brain tissue segmentation.

### 2.2 Image post-processing

Three reconstruction modes were tested for collected data: 1) k-space data reconstruction according to that described by Luo et al. [1], 2) vendor’s adaptive combination mode (ACM) [11,12] and 3) vendor’s sum-of-squares mode (SSM). Each sequence produced both k-space data and coil-combined image space DICOM data (ACM or SSM).

Luo et al. [1] started data processing from complex k-space data. However, the signal decay model can be separated to magnitude and phase part, which allows fitting a signal model to magnitude and phase images separately. This enables the use of DICOM data for signal decay fits. Our goal here was to test how combination mode affects the synthetic contrasts created from signal fit data.

An in-house program with MATLAB (MathWorks, Inc., Natick, MA, USA) was created to perform the post-processing for both k-space and DICOM data. First, k-space data were read with MATLAB functions by Philipp Ehses [13], and this data was then reconstructed to image space. Coil-channel combination was done with a sum-of-squares method, where phase offset was eliminated by setting phase to zero at the first TE in every channel.

The image generation was similar for all data after channel combination. The least squares method was applied to fit logarithmic magnitude data voxel-wise to a mono-exponential decay model, where fit parameters represented synthetic T1-weighted (T1w) image and quantitative T2* values. Signal phase data were similarly fitted to a linear decay model. Other synthetic images were created from these basic contrasts as described by Luo et al. [1]. We generated synthetic SWI-like and GEPCI-SWI images, their minimum intensity projections (mIP), T2*-SWI images, contrast enhanced T1w (T1f) images, and fluid suppressed T2* (FST2*) images, which remind fluid attenuation inversion recovery contrasts.

### 2.3 Image quality assessment

The impact of the reconstruction methods and acquisition choices was assessed by evaluating the changes on decay model fits, T2* values, image contrasts and phantom image quality tests.

To assess the accuracy of the decay model fits at the creation of synthetic contrasts, we calculated the root-mean-square error (RMSE) of magnitude and phase data fits for each voxel within the brain. A small RMSE value indicates a good fit and therefore a reliable fitting parameter estimate. In phantom measurements signal magnitude fit RMSE was investigated in a cylindrical volume of interest (VOI) in a uniform signal area with a radius of 40 mm and a height of 10 mm.

Median values of quantitative T2* were calculated to assess the approximate equivalence to literature. Median values were used instead of mean values, as they are not as prone to outliers. Voxel-wise median differences of T2* values (ΔT2*=T2*_ACM_ -T2*_k-space_) were calculated to gain insight into how the channel combination mode affects the quantitative values inside brain or VOI. ΔT2* were also calculated for each imaging sequence with different partial Fourier factors in comparison to the similar acquisition without the partial Fourier.

The measured magnitude images at the first TE were used for phantom image quality testing. Image quality assessment of the ACR phantom was performed with a semi-automatic in-house MATLAB program [14] according to phantom vendor instructions [10]. These tests included percent integral uniformity (%), slice thickness accuracy, and low-contrast object detectability, which was performed as a visual detection test of 40 low-contrast spokes at four inserts. Signal-to-noise ratios (SNR) were calculated from mean phantom and background signals [15].

In the patient studies, the T13D-images were segmented into white matter (WM), gray matter (GM), and cerebrospinal fluid (CSF) with the Statistical Parametric Mapping (SPM12) segmentation tool [16]. The segmented images were rigidly registered to the GEPCI-image space using FSL FLIRT [17,18]. To assess the image quality of synthetic brain images we calculated the relative WM/GM contrast and contrast-to-noise ratio (CNR). The relative WM/GM contrast was calculated for all synthetic patient images as the proportion of median WM and GM values. CNR was calculated for synthetic T1w images as absolute contrast per noise. We used the difference of median WM and GM as the absolute contrast, and the standard deviation (SD) of background voxels as the noise value. Voxels closer than 10 mm from the head or any of the image borders were excluded from the background.

Volunteer images were processed by brain extracting the synthetic T1w images with an FSL Brain Extraction Tool [19]. Then a threshold was used as a straightforward way to exclude robustly the CSF voxels from the images.

## 3 Results

### 3.1 Reconstruction methods

In the phantom measurements, the difference of the magnitude signal-fit RMSE were negligible, and the image quality measurements were not discernably affected by the used reconstruction mode (Table 1). We observed that ΔT2* was more dependent on the slice thickness than the reconstruction mode, and that differences were larger between two adjacent measurements with k-space reconstruction than between different reconstructions. The measurements with 5 mm slice thickness had decreased SNR and uniformity, and clearly increased T2* values. This is due to plastic phantom walls, which create Gibbs ringing (Supplementary File 1).

**Table 1:** ACR MRI phantom median T2* values and image quality results with different reconstruction modes.

Slice [mm]	Reconstruction a	Median T2* [ms]	Median ΔT2* b [ms]	SNR	Uniformity [%]	Thickness [mm]	Contrast c /40	Data
1	k-space	121.1	–	36.4	82.2	–	40	6.0 GB
	SSM	121.3	0.2	36.8	82.3	1.6	40	460 MB
2	k-space	118.3	–	37.8	82.5	2.7	37	3.0 GB
	k-space	120.7	2.4	36.3	82.5	2.5	39	3.0 GB
	SSM	118.9	0.5	37.8	82.7	2.8	37	230 MB
	ACM	119.9	- 0.9	37.5	82.5	2.7	38	230 MB
5	k-space	126.0	–	24.5	62.3	5.3	25	3.3 GB
	k-space	129.6	3.0	20.0	70.5	5.6	30	1.1 GB
	SSM	125.5	1.8	25.2	62.5	5.9	25	95 MB
	ACM	131.3	3.9	20.5	71.3	4.9	29	95 MB

a SSM and ACM are vendor’s sum of squares combination and adaptive combination mode respectively.

b ΔT2* is voxel-wise difference to the corresponding k-space data reconstruction.

c Low contrast detectability: visual inspection of the total number of visible spokes in four inserts (maximum 40).

**Fig. 1:**
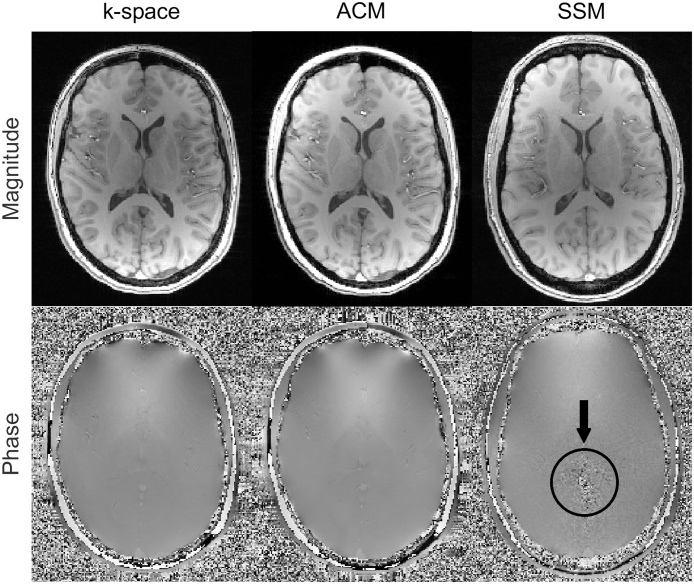
Comparison of magnitude (top row) and phase data (bottom row) from different reconstruction modes: k-space data reconstruction (left), vendor’s DICOM data with adaptive combination mode (ACM) (middle) and with sum-of-squares mode (SSM) (right). ACM data and k-space data come from the same acquisition. The SSM phase artifact is marked with a black circle and arrow. Other images are artifact free.

Brain images created from k-space data or with ACM had no visible artefacts (Fig. 1, left and middle columns). However, in the SSM phase images an artefact was observed (Fig. 1, right bottom), and these images were not analyzed further. This artefact is caused by the lack of phase-offset adjustment between different channels, and it would prevent the adequate production of all SWI-kind synthetic contrasts.

**Fig. 2:**
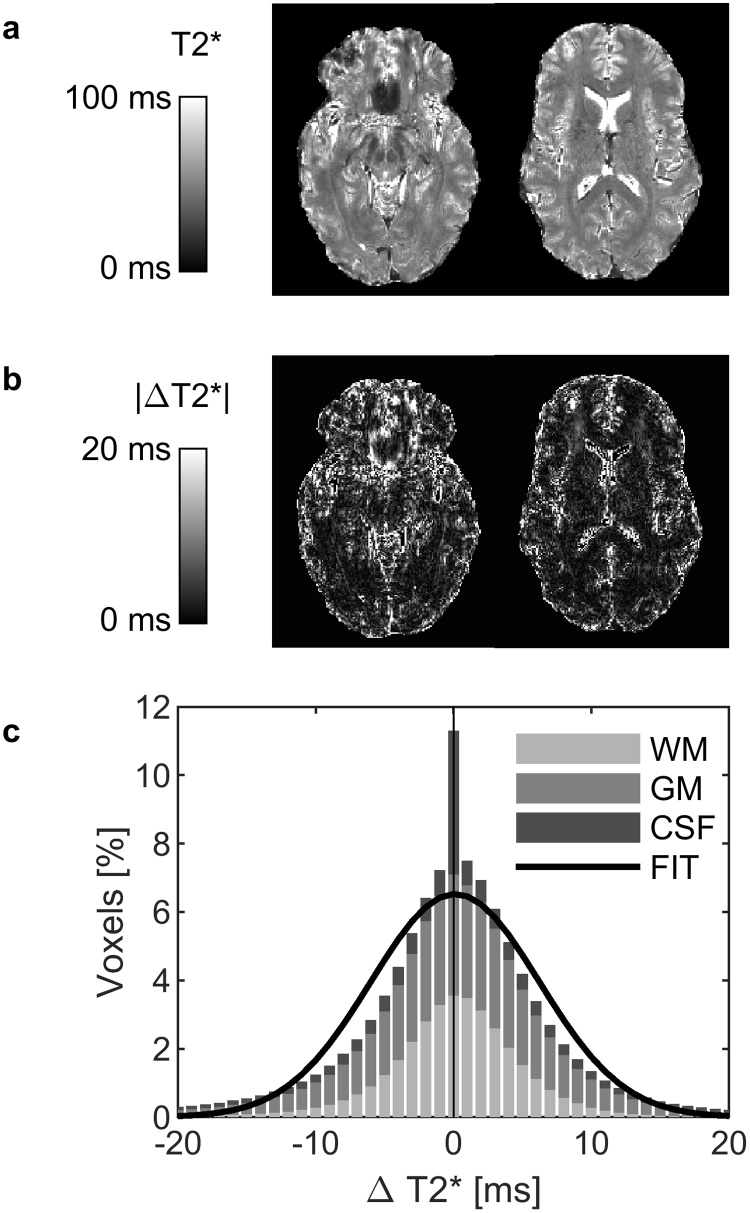
T2* differences between reconstruction modes. (a) Example slices of the T2* maps of one patient. (b) The magnitude of voxel-wise T2* differences (ΔT2*) between vendor’s adaptive combination mode and k-space reconstruction. (c) Distribution of ΔT2* in white matter (WM), grey matter (GM) and cerebrospinal fluid (CSF). Fit represents Gaussian distribution fit to WM and GM data. Quantitative scales are in milliseconds.

An example of the T2* image (Fig. 2a) and an absolute difference image between ACM and k-space data reconstruction (Fig. 2b) indicated that voxels with high absolute difference were mainly in CSF or located near vessels or high susceptibility areas in GM. The examination of CSF T2* values was not continued, because they were not reliable. The distribution of voxel-wise ΔT2* values (Fig. 2c) denoted that the distribution of WM and GM values does not follow normal distribution, and therefore median metrics can better describe the differences between acquisition choices or reconstruction methods. Distribution of ΔT2* values had a slight skewness for the positive side, meaning that ACM produced little higher T2* values than k-space data approach.

The average and SD of the median T2* values over all patients with k-space data reconstruction were 48.0 ± 2.1 ms for WM and 54.5 ± 3.1 ms for GM. Similar values for ACM were 48.6 ± 1.6 ms and 55.2 ± 2.3 ms. The average of the median ΔT2* over all patients was 2.9 ± 0.7 ms for WM and 4.5 ± 0.6 ms for GM. We also found that 93% of WM voxels and 78% of GM voxels had ΔT2* less than 10 ms.

**Fig. 3:**
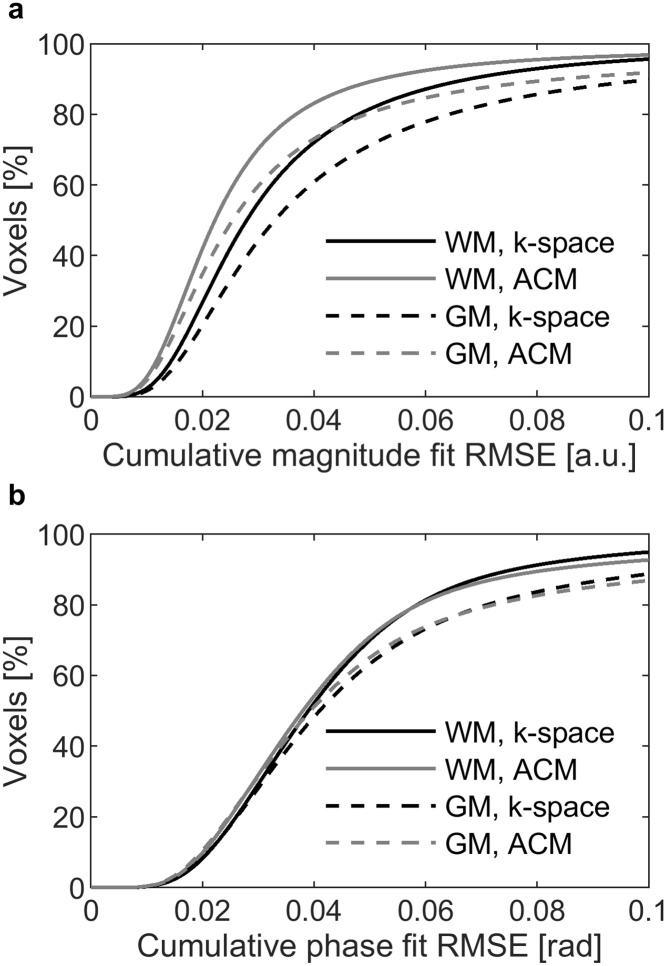
Cumulative histograms of signal fit error of patients. Root mean square error (RMSE) of (a) signal magnitude fit in arbitrary units (a.u.), and (b) signal phase fit in radians (rad). RMSE for white matter (WM) and grey matter (GM) with vendor’s adaptive combination mode (ACM) and k-space reconstruction are plot separately.

**Fig. 4:**
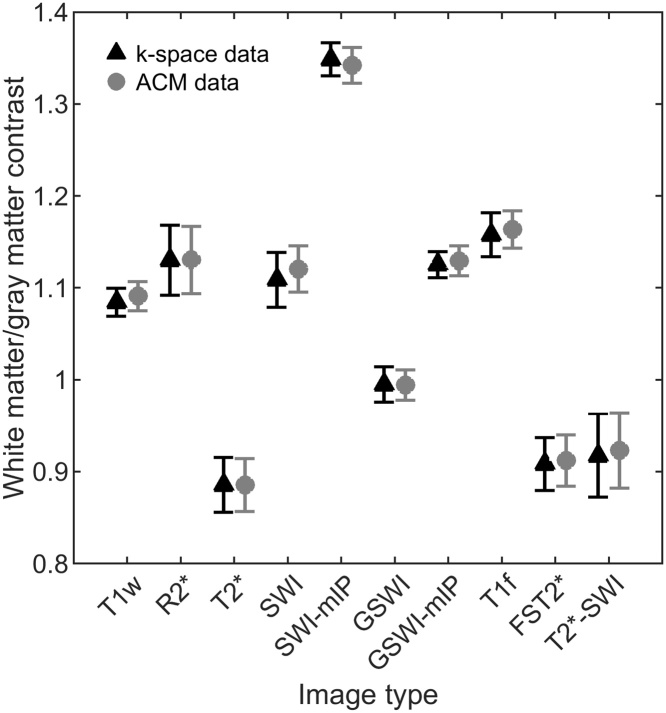
Mean and standard deviation of the white matter/gray matter contrast of all 8 patients. Results for 10 synthetic contrasts with reconstructions from k-space data and with vendor’s adaptive combination mode (ACM) are shown. Images are T1-wighted (T1w), R2*, T2*, susceptibility weighted image (SWI) and SWI without T1-weighting (GSWI), their minimum intensity projections (mIP), contrast enhanced T1-image (T1f), fluid suppressed T2* (FST2*) and FST2* based SWI (T2*-SWI).

Signal magnitude (Fig. 3a) and phase fit RMSE (Fig. 3b) were smaller in WM than in GM, and the magnitude RMSE seemed to be slightly higher for the k-space data reconstruction. Median relative contrast and distribution varied with different image types (Fig. 4), but the differences between reconstruction modes were small. Especially, quantitative R2* and T2* values showed no dependence on the combination mode. GEPCI-SWI images had the worst contrast (closest to 1), but this could be improved with minimum intensity projection. Contrast enhanced T1f images had also better contrast than unprocessed synthetic T1w images. CNR from k-space reconstructed T1w-image was 6.6 and from ACM T1w-image 7.2.

### 3.2 Partial Fourier

**Fig. 5:**
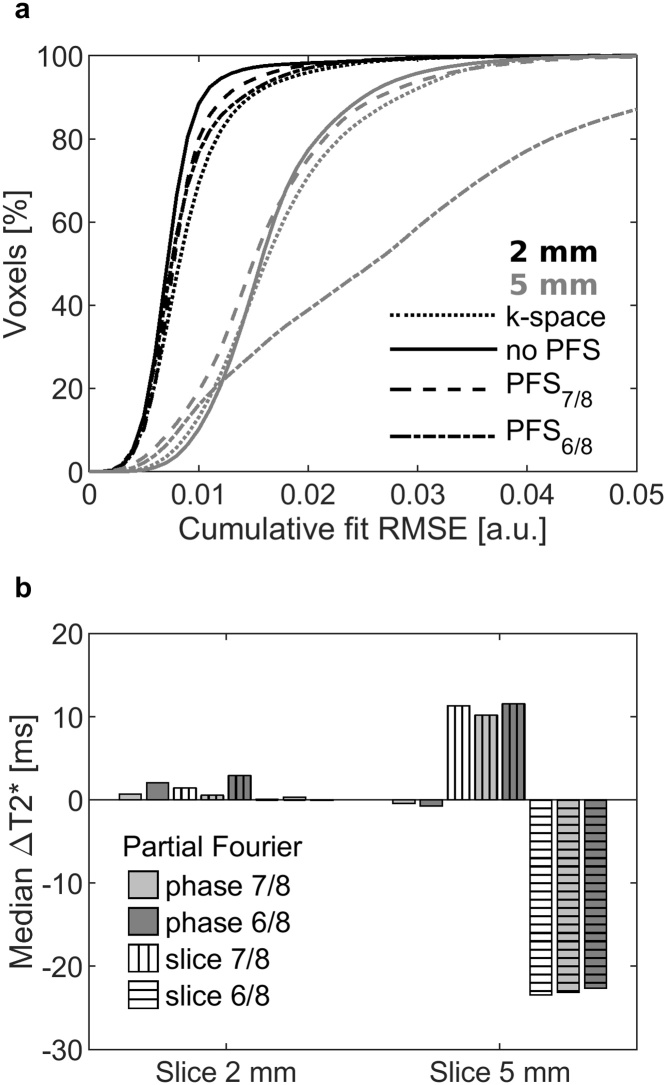
(a) Cumulative signal magnitude fit root mean square error (RMSE) and (b) median T2* difference (ΔT2*) of phantom measurements. Slice thickness 2 millimeter and 5 millimeter, as well as acquisition with different factors of partial Fourier imaging in phase (PFP) and slice direction (PFS) were tested. Median ΔT2* values are calculate relative to acquisition with the same slice thickness and no partial Fourier applied.

Acquisitions with PFS increased the ΔT2*, overestimated the slice thickness, and decreased visual low-contrast detection (Table 2). Cumulative RMSE histograms with different acquisition parameters and slice thickness (Fig. 5a) showed that acquisitions with 5 mm slice thickness had higher fit-errors, and acquisition with PFS_6/8_ and 5 mm slice thickness had the highest RMSE.

**Table 2:** ACR MRI phantom median T2* values and image quality results with different acquisition parameters.

Slice [mm]	Parameters a	Median T2* [ms]	Median ΔT2* b [ms]	SNR	Uniformity [%]	Thickness [mm]	Contrast^c^/40	Scan time [min:s]
2	Original	118.9	–	37.8	82.7	2.8	37	14:08
	PFP_7/8_	121.0	2.1	38.7	82.4	2.8	39	12:22
	PFS_7/8_	120.2	1.5	38.0	82.4	3.1	38	12:22
	PFP_6/8_	119.6	0.7	38.5	82.5	2.7	39	10:37
	PFS_6/8_	118.6	0.1	36.3	82.6	3.5	33	10:37
	PFP_7/8_ + PFS_7/8_	119.3	0.6	36.3	82.4	3.1	37	10:50
	PFP_7/8_ + PFS_6/8_	118.9	0.4	36.4	82.8	3.6	31	9:17
	PFP_6/8_ + PFS_7/8_	121.8	3.0	36.3	82.4	3.1	39	9:17
	PFP_6/8_ + PFS_6/8_	118.4	- 0.03	36.3	82.6	3.1	39	7:58
5	Original	131.3	–	20.5	71.3	4.9	29	
	PFP_7/8_	131.3	- 0.4	20.9	71.2	5.3	26	
	PFS_7/8_	145.3	11.3	21.3	71.6	5.8	15	
	PFP_6/8_	130.9	- 0.7	20.9	71.4	5.4	30	
	PFS_6/8_	104.9	- 23.4	20.7	71.6	6.2	7	
	PFP_7/8_ + PFS_7/8_	145.1	10.2	21.1	71.7	5.4	18	
	PFP_7/8_ + PFS_6/8_	105.6	- 23.1	20.7	71.7	6.2	8	
	PFP_6/8_ + PFS_7/8_	146.1	11.6	21.3	71.6	5.7	26	
	PFP_6/8_ + PFS_6/8_	106.1	- 22.6	20.7	71.6	6.0	13	

a PFP and PFP are partial Fourier techniques in phase and slice direction respectively.

b ΔT2* is the voxel-wise difference to the corresponding measurement with the original acquisition settings.

c Low contrast detectability: visual inspection of total number of visible spokes in four inserts (maximum 40).

The median ΔT2* (Fig. 5b) of 2 mm slice measurements were mostly under 1 ms, and no higher than 3 ms regardless of the reconstruction. However, acquisitions with 5 mm slice thickness depended on the PFS k-space coverage, so that measurements with no PFS had median ΔT2* under 1 ms, but with PFS_7/8_ the magnitude of ΔT2* was over 10 ms and with PFS_6/8_ over 20 ms. Similar over- and underestimation was also seen with the absolute T2* values.

**Fig. 6:**
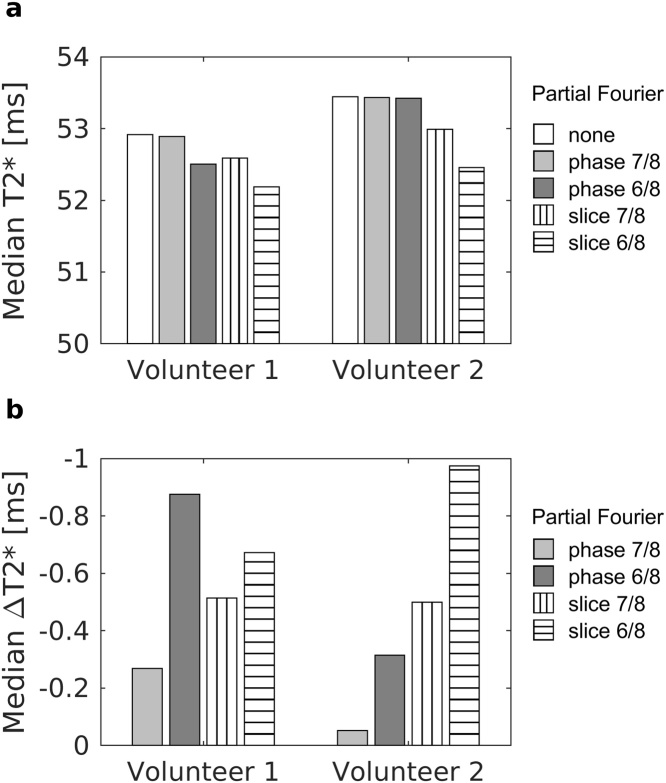
(a) Median T2* and (b) voxel-wise ΔT2* of two healthy volunteers imaged with different Partial Fourier settings. Volunteer 1 was imaged with a 12-channel head coil and volunteer 2 with a 32-channel head coil. Quantitative scales are in milliseconds.

In volunteer studies the use of partial Fourier techniques or the number of the channels in the head coil had only a small effect on the T2* values (Fig. 6a), although acquisitions with PFS resulted with slightly decreased T2*. Voxel-wise ΔT2* differences between original acquisition and measurements with different k-space coverage of PFP and PFS were all under 1 ms (Fig. 6b). Magnitude fit errors were also slightly smaller with a 32-channel coil (volunteer 2) than with a 12-channel coil (volunteer 1), but no difference in phase data was seen.

## 4 Discussion

The comparison of coil combination modes shows that SSM reconstruction should not be used for phase data acquisitions. This was not seen in phantom measurements, thus this type of testing should be performed *in vivo* or on a phantom with similar tissue behavior as in brain. The changes in reconstruction should be handled with caution, especially when quantitative data are acquired. Our experiment included only one scanner, but similar pitfalls may exist with scanners from other vendors.

In phantom measurements with 2 mm slice thickness ΔT2* was smaller between different reconstruction modes, than between two adjacent acquisitions with k-space reconstruction. In patient studies the results suggest that ACM reconstruction method preserves the quantitative nature of T2* values in WM and GM, since the median ΔT2* were less than 5 ms. Also, no clear change in median T2*, signal fit RMSE, WM/GM contrast, or CNR was seen. Largest ΔT2* differences in GM mostly occurred near susceptibility artifacts, or pulsating vessels and ventricles. The T2* values in proximity to vessels or susceptibility artifacts are unreliable even with the reconstruction of k-space data, since pulsation, flow, and partial volume artifacts can corrupt these voxels. Segmentation was performed on the T13D-image, which was then registered to the GEPCI-image space. This can cause partial volume artifacts and inaccuracies in the automatic segmentation.

CNR was determined only for T1-weighted GEPCI-images, because the background in synthetic images does not describe noise correctly. This can be seen for example in the phase images of Fig. 1 (bottom row), which suggest that at least all synthetic contrasts using phase data have unphysically high background variation.

The T2* values reported in the literature vary in the range 45–54 ms for WM [[20], [21], [22], [23], [24], [25]] and 42–75 ms for GM [20,21,24,25], and our measurements are in line with those observations. The differences in T2* between coil-channel combination modes were smaller than the variation between different studies in the literature [[20], [21], [22], [23], [24]], or the differences between frontal and occipital regions [20,23]. R2* (1/T2*) values of the brain produced with GEPCI post-processing have been shown to agree with literature values [26,27]. We did not correct the T2* for trough-slice dephasing effect [24], or other factors possibly affecting T2*, as the main aim was to evaluate the differences between acquisition and reconstruction techniques rather than accurate T2*-values. Signal fits for T2* evaluation were performed with least squares fitting, which is known to be sensitive to noise [28], but it is widely used and gives fast and robust parameter estimates. More optimal fitting algorithms could be investigated in a separate study.

The use of PFS caused variation in T2* values, increase of ΔT2* and worse signal fit in phantom measurements. Voxel-wise ΔT2* values with different imaging parameters are not measured from the same acquisition, and therefore subject movements can affect these values. Our phantom data suggest that PFS techniques might not be optimal for GEPCI, although explicit differences in brain data were not seen. Our experiments were limited to scrutinizing only the partial Fourier techniques, and additional acquisition parameters could be further studied to find a more optimal combination.

Quantitative T2* values, WM/GM-contrast, or signal-fit error comparisons might not describe the clinical image quality completely. Also, there is no quality reference metric for the ACR phantom to study the signal phase. In future, studies with a relaxation phantom could be used to compare quantitative T2* values. Computer simulations could also be used to characterize how partial Fourier techniques affect the image quality of synthetic contrasts.

## 5 Conclusion

Our results indicate that GEPCI post-processing technique can be used to produce synthetic contrasts from ACM combined DICOM data, instead of k-space data. The quantitative T2* values of WM and GM are preserved in this change. Synthetic images can be generated offline on an auxiliary computer with a simplified reconstruction pipeline, which would result in reduced data storage and transfer demands without significant sacrifices in image quality. To speed up the acquisition, partial Fourier technique is more recommended in phase than slice direction.

## Conflict of interest

Authors declare no conflicts of interest.
